# Anxiety in response to the climate and environmental crises: validation of the Hogg Eco-Anxiety Scale in Germany

**DOI:** 10.3389/fpsyg.2023.1239425

**Published:** 2023-09-21

**Authors:** Stephan Heinzel, Mira Tschorn, Michael Schulte-Hutner, Fabian Schäfer, Gerhard Reese, Carina Pohle, Felix Peter, Michael Neuber, Shuyan Liu, Jan Keller, Michael Eichinger, Myriam Bechtoldt

**Affiliations:** ^1^Institute of Psychology, Department of Educational Sciences and Psychology, TU Dortmund University, Dortmund, Germany; ^2^Department of Education and Psychology, Freie Universität Berlin, Berlin, Germany; ^3^Social and Preventive Medicine, Department of Sports and Health Sciences, University of Potsdam, Potsdam, Germany; ^4^Sustainable Development, Darmstadt University of Applied Sciences, Darmstadt, Germany; ^5^Klimabildung e.V., Bochum, Germany; ^6^Department of Psychology, RPTU Kaiserslautern Landau, Campus Landau, Landau, Germany; ^7^Department of School Psychology, State School Administration of Saxony-Anhalt, Halle (Saale), Germany; ^8^Center for Technology and Society, Technical University of Berlin, Berlin, Germany; ^9^Department of Psychiatry and Psychotherapy (CCM), Charité – Universitätsmedizin Berlin, Berlin, Germany; ^10^Center for Preventive Medicine and Digital Health, Medical Faculty Mannheim, Heidelberg University, Mannheim, Germany; ^11^Institute of Medical Biostatistics, Epidemiology and Informatics, University Medical Center of the Johannes Gutenberg-University Mainz, Mainz, Germany; ^12^Department of Management, EBS Universität für Wirtschaft und Recht, Oestrich-Winkel, Germany

**Keywords:** environmental crisis, climate crisis, climate change, eco-anxiety, climate anxiety, Hogg Eco-Anxiety Scale

## Abstract

**Background:**

As the climate and environmental crises unfold, eco-anxiety, defined as anxiety about the crises’ devastating consequences for life on earth, affects mental health worldwide. Despite its importance, research on eco-anxiety is currently limited by a lack of validated assessment instruments available in different languages. Recently, Hogg and colleagues proposed a multidimensional approach to assess eco-anxiety. Here, we aim to translate the original English Hogg Eco-Anxiety Scale (HEAS) into German and to assess its reliability and validity in a German sample.

**Methods:**

Following the TRAPD (translation, review, adjudication, pre-test, documentation) approach, we translated the original English scale into German. In total, 486 participants completed the German HEAS. We used Bayesian confirmatory factor analysis (CFA) to assess whether the four-factorial model of the original English version could be replicated in the German sample. Furthermore, associations with a variety of emotional reactions towards the climate crisis, general depression, anxiety, and stress were investigated.

**Results:**

The German HEAS was internally consistent (Cronbach’s alphas 0.71–0.86) and the Bayesian CFA showed that model fit was best for the four-factorial model, comparable to the factorial structure of the original English scale (affective symptoms, rumination, behavioral symptoms, anxiety about personal impact). Weak to moderate associations were found with negative emotional reactions towards the climate crisis and with general depression, anxiety, and stress.

**Discussion:**

Our results support the original four-factorial model of the scale and indicate that the German HEAS is a reliable and valid scale to assess eco-anxiety in German speaking populations.

## 1. Introduction

The climate and environmental crises pose existential threats to human survival (Kates et al., 2012) and adversely affect mental health worldwide (Hickman et al., 2021; Corvalan et al., 2022). Catastrophic and more frequent extreme weather events as well as anticipated changes in living conditions lead to considerable anxiety and other emotions, even among people not yet adversely affected. In this context, eco-anxiety has been defined as anxiety about the climate and environmental crises’ devastating consequences for life on earth (Pihkala, 2020; Hogg et al., 2021). Eco-anxiety is considered an umbrella term and comprises “climate change anxiety,” i.e., anxiety specifically related to the anthropogenic climate change (Clayton, 2020; Clayton and Karazsia, 2020), as well as “anxiety about a multiplicity of environmental calamities, which may or may not be directly caused by climate change, including the elimination of entire ecosystems and plant and animal species, global mass pollution and deforestation” (Hogg et al., 2021, p. 3).

Expanding earlier conceptions of climate change and eco-anxiety primarily focused on affective symptoms (Searle and Gow, 2010; Helm et al., 2018), current measures acknowledge the multidimensionality of these constructs (Clayton and Karazsia, 2020; Hogg et al., 2021), including cognitions and behavioral impairments operationalized by items such as “unable to stop thinking about losses to the environment” or “difficulty sleeping.” The first validated multidimensional climate anxiety scale was developed by Clayton and Karazsia (2020) and comprised two dimensions: cognitive-emotional impairment (e.g., “I find myself crying because of climate change”) and functional impairment (e.g., “My concerns about climate change undermine my ability to work to my potential”). Despite advancements compared to earlier conceptions of climate change anxiety, the Climate Anxiety Scale has certain limitations. First, the scale focuses on the climate crisis as the sole cause of anxiety and disregards other devastating environmental calamities caused by human activity, such as deforestation or pollution. Second, the scale emphasizes different impairments caused by the climate crisis but does not capture the emotional experience of anxiety (Wullenkord et al., 2021). Third, Wullenkord et al. (2021) were unable to replicate the factorial structure of the Climate Anxiety Scale of Clayton and Karazsia (2020) in a German sample and point to conceptual limitations. Supporting this finding, a recent synthesis of psychometric properties of the Climate Anxiety Scale and the Hogg Eco-Anxiety Scale (HEAS; Hogg et al., 2023) indicated that psychometric performance of the Climate Anxiety Scale was mixed and inconsistent (Larionow et al., 2022; Mouguiama-Daouda et al., 2022; Simon et al., 2022; Tam et al., 2023).

To expand previous work, Hogg and colleagues proposed the HEAS as an alternative measure to the Climate Anxiety Scale. Their validation studies (Hogg et al., 2021) yielded a four-factorial model, comprising affective symptoms, rumination, behavioral symptoms and anxiety of one’s personal impact on the planet, that supports the multidimensionality of eco-anxiety. Furthermore, they reported high internal consistency and moderate associations with general anxiety, depression, and stress.

The global adverse effects of the climate and environmental crises on mental health highlight the importance of making reliable and valid scales of eco-anxiety available in different languages to facilitate cross-country research. Since no validated eco-anxiety scale is currently available in German, the aim of this study was to translate the original English HEAS (Hogg et al., 2021) into German and to assess its psychometric properties. This included an examination of its internal consistency, factorial structure and associations with general anxiety, depression, and stress as well as various emotional reactions to the climate crisis.

## 2. Methods

We used a two-step approach in this study. In a first step, we translated the original English HEAS into German using standardized guidance (Dorer, 2018). In a second step, we conducted a cross sectional study to assess the psychometric properties of the German HEAS. The study was approved by the local ethics committee of Freie Universität Berlin, Germany (No 036/2021 and Amendments 020/2022 and 023/2022 for samples 1 and 2) and by the local ethics committee at University of Potsdam, Germany (No 51/2022 for sample 3). We obtained written informed consent from all participants.

### 2.1. Translation of the original English HEAS into German

We translated the original English HEAS (Hogg et al., 2021) into German using the TRAPD approach (translation, review, adjudication, pre-test, documentation) as recommended by the European Social Survey (Dorer, 2018). First, three German native speakers fluent in English (SH, MS-H, and FP) independently translated the scale into German. Second, the three versions were discussed in the research team and integrated, yielding a pre-test version of the scale. Third, following the back-translation approach (Brislin, 1970), two researchers fluent in English (C2 level) and not involved in our study translated the scale back into English. Based on the back-translations, we made minor adjustments to the German pre-test version. Forth, we conducted a pilot survey with 33 participants to assess the comprehensibility of the scale. Given good comprehensibility of all items in the pilot survey, we did not apply further changes to the German HEAS. The English and German items of the HEAS are reported in Table 1. The protocol of the translation can be requested from the corresponding author.

**Table 1:** Original English items of the Hogg Eco-Anxiety Scale (HEAS) and their German translation.

Original English HEAS	German translation of the HEAS
Over the last 2 weeks, how often have you been bothered by the following problems, when thinking about climate change and other global environmental conditions (e.g., global warming, ecological degradation, resource depletion, species extinction, ozone hole, pollution of the oceans, deforestation)?	Wie oft fühlten Sie sich im Verlauf der letzten 2 Wochen durch die folgenden Beschwerden beeinträchtigt, wenn Sie über den Klimawandel und andere globale Umweltbedingungen nachdachten (z.B. globale Erwärmung, Umweltzerstörung, Ressourcenerschöpfung, Artensterben, Ozonloch, Verschmutzung der Ozeane, Abholzung?)
1. Feeling nervous, anxious or on edge	1. Nervosität, Ängstlichkeit oder Anspannung
2. Not being able to stop or control worrying	2. Nicht in der Lage sein, Sorgen zu stoppen oder zu kontrollieren
3. Worrying too much	3. Übermäßige Sorgen
4. Feeling afraid	4. Gefühl der Angst
5. Unable to stop thinking about future climate change and other global environmental problems	5. Nicht in der Lage sein, das Nachdenken über den zukünftigen Klimawandel und andere globale Umweltprobleme zu stoppen
6. Unable to stop thinking about past events related to climate change	6. Nicht in der Lage sein, das Nachdenken über vergangene Ereignisse zu stoppen, die mit dem Klimawandel zusammenhängen
7. Unable to stop thinking about losses to the environment	7. Nicht in der Lage sein, das Nachdenken über Schäden für die Umwelt zu stoppen
8. Difficulty sleeping	8. Schwierigkeiten zu schlafen
9. Difficulty enjoying social situations with family and friends	9. Schwierigkeiten soziale Situationen mit Familie und Freund*innen zu genießen
10. Difficulty working and/or studying	10. Schwierigkeiten zu arbeiten und/oder zu lernen
11. Feeling anxious about the impact of your personal behaviors on the earth	11. Besorgnis über die Auswirkungen Ihrer persönlichen Verhaltensweisen auf die Erde
12. Feeling anxious about your personal responsibility to help address environmental problems	12. Besorgnis über Ihre persönliche Verantwortung beim Angehen von Umweltproblemen
13. Feeling anxious that your personal behaviors will do little to help fix the problem	13. Besorgnis, dass Ihr persönliches Verhalten wenig zur Lösung des Problems beitragen wird
Response scale: 0 = not at all, 1 = several of the days, 2 = over half the days, 3 = nearly every day.	Antwortalternativen: 0 = Überhaupt nicht, 1 = An einzelnen Tagen, 2 = An mehr als der Hälfte der Tage, 3 = Beinahe jeden Tag

HEAS comprises the following four subscales: affective symptoms (true mean of items 1–4), rumination (true mean of items 5–7), behavioral symptoms (true mean of items 8–10), anxiety about personal impact (true mean of items 11–13).

### 2.2. Participants and data collection

We tested the German HEAS with 486 participants (121 male/357 female/8 diverse) in Germany (age *M* [*SD*] = 29.43 [10.63], median: 26 years, range: 18–73). To reach an adequate sample size for our planned analyses, we used three different recruitment approaches: 158 participants (students) were recruited via online advertisements and mailing-lists at 40 German universities (sample 1). One hundred and ninety-six participants (students and university staff) were recruited at Freie Universität Berlin via flyers, posters, and emails (sample 2) and 132 participants (students) at University of Potsdam via an online recruitment system for university students and online advertisement (sample 3). Using a link/QR code on the study invitation, participants accessed and completed an online survey implemented in the survey software *Unipark* (Version 21.2, QuestBack GmbH, Oslo, Norway).

### 2.3. Measures

#### 2.3.1. HEAS

The Hogg Eco-Anxiety Scale (HEAS; Hogg et al., 2021) comprises 13 items (see Table 1 for the German items) and is intended to measure four dimensions of anxiety related to the climate and environmental crises: affective symptoms, rumination, behavioral symptoms, and anxiety about one’s negative impact on the planet. For each item, the frequency during the past 2 weeks was self-rated on a 4-point Likert scale (0 = not at all, 1 = several of the days, 2 = over half the days, 3 = nearly every day). The validation study of the original English HEAS confirmed the postulated four-factorial structure, with all subscales being internally consistent (all Cronbach’s alphas >0.82).

#### 2.3.2. DASS-21

In accordance with Hogg et al. (2021), we used the Depression Anxiety Stress Scale 21 (DASS-21; Lovibond and Lovibond, 1995) to assess associations between eco-anxiety and general depression, anxiety, and stress. The DASS-21 measures self-reported symptoms during the past 2 weeks on a 4-point Likert scale (from 0 “did not apply to me at all” to 3 “applied to me very much or most of the time”) with higher scores indicating higher symptom burden.

#### 2.3.3. Emotional reactions in response to the climate crisis

To investigate associations between eco-anxiety and other emotional reactions towards the climate crisis, we included 18 positive and negative emotions, each assessed with a single item based on work by Hickman et al. (2021). Participants were asked to rate the current strength of each emotion when thinking about the climate crisis on a 5-point Likert scale ranging from “not at all” to “extremely.”

All participants completed the German HEAS and items on emotional reactions towards the climate crisis. Participants of sample 1 additionally completed the DASS-21. To reduce participant burden and to achieve high response rates necessary for the planned analyses, sample 2 and 3 did not complete the DASS-21. Respondents received no financial compensation for participating in the study.

### 2.4. Data analysis

We calculated Cronbach’s alpha for all HEAS subscales to estimate internal consistency. To explore concurrent and discriminant validity, we investigated associations with the DASS-21 and emotional reactions towards the climate crisis. Comparable to Hogg et al. (2021), we fitted a multiple linear regression model to assess unique associations between the DASS-21 anxiety subscale and each HEAS subscale while controlling for the remaining HEAS subscales. To test unique associations of emotional reactions with the HEAS affective symptoms subscale, we fitted a second multiple linear regression model. Furthermore, we calculated bivariate Pearson correlations between HEAS subscales and DASS-21 subscales as well as emotional reactions towards the climate crisis.

We performed a Bayesian confirmatory factor analysis (CFA) to examine the factorial structure of the German HEAS. Bayesian CFA, unlike conventional CFA, offers certain benefits: Bayesian CFA employs probabilistic methods to effectively estimate parameters even when sample sizes are modest, mitigating issues associated with statistical power and enhancing the robustness of findings. Another notable feature of Bayesian CFA is its capacity to incorporate existing knowledge or beliefs into the analysis through “informed priors.” This means that researchers can introduce relevant information about parameter values before analyzing the data. Such *a priori* knowledge enhances the precision of parameter estimates and refines the accuracy of model outcomes. Furthermore, Bayesian CFA offers a more versatile approach for modeling cross-loadings of items on latent factors. Unlike conventional CFA, which only allows for substantial loadings of items on their respective factors, Bayesian CFA accounts for the possibility that items may have subtle yet meaningful relationships with other factors (near-zero loadings). This permits a more nuanced understanding of the relationships between variables, resulting in a more comprehensive representation of the underlying constructs (Depaoli, 2021). These attributes collectively establish Bayesian CFA as a valuable analytical tool, extending researchers’ capabilities to address challenges posed by limited samples, to leverage existing knowledge, and to effectively model complex relationships within the data.

We chose Bayesian CFA to incorporate information from prior work including data about the factorial structure and item loadings reported by Hogg et al. (2021) in our analysis. Considering this knowledge in the estimation process enabled us to include pre-existing information about the model parameters and update these assumptions (Depaoli and van de Schoot, 2017). Even small to moderate samples are sufficient to obtain accurate results if, as in this case, there is prior knowledge (Depaoli and van de Schoot, 2017). Model fit was assessed using posterior predictive checks, which compared the observed data with the estimated model, i.e., the posterior predictive distribution (Muthén and Muthén, 2017).

The statistics program M*plus* (version 8, Muthén and Muthén, 2017) produces a confidence interval for the posterior predictive checks, which, if they include zero indicate that the hypothesized model structure adequately fits the observed data. The posterior predictive (PP) value of *p* indicates the proportion of replicated data that exceeds the original data. Low PP values of *p* indicate poor fit. Models with values <0.10 should be rejected, whereas values around 0.5 indicate excellent model fit (Cain and Zhang, 2019).

We compared a four-factorial model in which the main loadings of the items on their hypothesized factors were specified to a four-factorial model in which near-zero cross-loadings of items from the three factors rumination, behavioral symptoms and anxiety about personal impact were allowed on the affective symptoms factor (Muthén and Asparouhov, 2012). We did this to account for the fact that in a principal component analysis by Hogg et al. (2021), the affective symptoms factor was found to explain 50% of the item variance. To evaluate the assumption of minor cross-loadings of items from the rumination, behavioral symptoms and anxiety about personal impact factor on affective symptoms, we checked the prior-posterior predictive (PPP) value of *p* (Asparouhov and Muthén, 2017). The PPP value of *p* differs from the PP value of *p*: The PPP value of *p* is suitable for testing whether the assumptions of near-zero priors with small variances hold. This way, individual parameters are tested rather than the fit of the overall model. If the PPP value of *p* is close to zero, the hypothesis that these cross-loadings are minor is rejected, which may indicate model misspecification. Any cross-loadings that are not minor would contradict the intended clear assignment of items to their respective factors. In addition, we compared the four-factorial solution to three-, two-and one-factorial models where rumination, behavioral symptoms and anxiety about personal impact were merged with the affective symptoms factor, respectively.

The reporting of the analyses follows the recommendations by Depaoli and van de Schoot (2017). The CFA was performed using Bayesian estimation in M*plus* (version 8; Muthén and Muthén, 2017). Two Markov chains were implemented for each parameter. A Markov chain is a computational algorithm to iteratively approximate the model parameters. Its characteristics imply that each new parameter value is conditional only on the preceding one, irrespective of the entire history of the chain. Through numerous iterations, Markov chains gradually converge towards a more accurate approximation of the true parameter values. To assess chain convergence, the Gelman and Rubin convergence diagnostic (Gelman and Rubin, 1992a,b) was implemented as described in the M*plus* manual with a stricter convergence criterion than the default setting (0.01 instead of 0.05). To establish stable calculations, we initiated a preliminary phase of 50,000 iterations (initial burn-in phase) without recording the results, followed by a fixed number of 50,000 iterations with recorded outcomes (postburn-in iterations). The Gelman and Rubin (1992a,b) diagnostic indicated that convergence was obtained with these fixed iterations for each of the two chains. Next, the trace plots for each model parameter were visually inspected. For each of the model parameters, both chains showed a constant mean and variance in the postburn-in portion of the chain. To further endorse convergence, we estimated the model again but with the number of burn-in and postburn-in iterations doubled (i.e., 200,000 iterations in total). Again, convergence was obtained and the model parameters were almost identical for all main factor loadings and factor covariances, i.e., the percent of relative deviation was less than 1%. However, this was not true for minor cross-loadings of items on the affective symptoms factor (see below). The magnitude of their factor loadings was substantially greater in the model with doubled iterations than in the original model, with a relative deviation of up to 300%. We therefore report the results of models with doubled iterations.

When implementing the informative priors for the main loadings of items on their respective factors, i.e., using the estimates from Table 5 in Hogg et al. (2021), we followed the recommendations by M*plus* and assumed that they followed a normal distribution. As Hogg and colleagues did not provide variances for the factor loadings, we conducted sensitivity analyses with different variance priors (0.001, 0.01, 0.1, 1.0 and 10) to test the robustness of our findings. We relied on the M*plus* default prior settings for error variances of items and the latent factor covariance matrix.

Recognizing the relatively infrequent use of Bayesian CFA, we also offer model fit indices of a conventional CFA to enhance interpretation. In evaluating model fit, we applied the following criteria: *χ*^2^/*df* ≤ 2, Comparative Fit Index (CFI; Bentler, 1990) ≥0.95, Tucker Lewis Index (TLI; Tucker and Lewis, 1973) ≥0.95, Root Mean Square Error of Approximation (RMSEA; Steiger, 1980) ≤0.05, and Standardized Root Mean Squared Residual (SRMR; Hu and Bentler, 1999) ≤0.08. However, since conventional CFA does not permit the modeling of near-zero loadings for items 5–13 on the affective symptoms factor, the Bayesian model is more complex and not entirely congruent with the four-factorial conventional CFA.

## 3. Results

### 3.1. Internal consistency of the German HEAS

The HEAS subscales affective symptoms (*M* [*SD*] = 0.69 [0.60]), rumination (*M* [*SD*] = 0.60 [0.67]), and anxiety about personal impact (*M* [*SD*] = 1.20 [0.70]) showed good internal consistency (Cronbach’s alphas = 0.83; 0.86; 0.83, respectively). The internal consistency of the subscale behavioral symptoms (*M* [*SD*] = 0.33 [0.50]) was acceptable (Cronbach’s alpha = 0.71).

### 3.2. Structural validity of the German HEAS

Overall, model fit was best for the four-factorial model with variance priors of 0.1 (results for models with different variance priors are available upon request). The model fit was acceptable based on a PP value of *p* of 0.187, and the four-factorial model showed better fit than the three-, two-, and one-factorial solutions where items loaded onto the affective symptoms factor instead of their content-specific factors. Furthermore, the Bayesian posterior predictive checking utilized *χ*^2^ likelihood ratio tests to compare the observed-data test statistic with the replicated-data test statistic. Since its 95% confidence interval included zero, this result indicated that there was no significant difference between the observed-data *χ*^2^ values and the replicated-data *χ*^2^ values. Finally, the PPP value of *p* of 0.902 was excellent and suggested that the assumption of near-zero cross-loadings in the four-factorial model was valid. The findings are presented in Table 2.

**Table 2:** Model fit comparisons.

	Posterior predictive check 95% CI		Posterior predictive *p*-value	Prior posterior predictive *p*-value
	Lower 2.5%	Upper 2.5%		
4-Factorial model, informed priors, no cross-loadings	−20.596	62.997	0.166	0.987
4-Factorial model, informed priors, near zero cross-loadings	−23.007	61.230	0.187	0.902
4-Factorial model, diffuse priors, near zero-cross-loadings	−19.774	64.523	0.151	0.000
3-Factorial model, informed priors and near-zero cross-loadings	38.02	133.949	0.000	0.973
2-Factorial model, informed priors and near-zero cross-loadings	61.853	163.564	0.000	0.906
1-Factorial model, informed priors	212.347	331.619	0.000	0.424

*N* = 486; CI, confidence interval.

Model fit indices from conventional frequentist CFA support the conclusion of good model fit with exception for the *χ*^2^ value: *χ*^2^/*df* = 2.45 (144.47/59), *p* ≤ 0.001, CFI = 0.983, TLI = 0.977, RMSEA = 0.055 (90% CI, 0.043–0.066), and SRMR = 0.024.

Table 3 displays the factor loadings of items in the four-factorial model with minor cross-loadings of items from the rumination, behavioral symptoms and anxiety about personal impact factor on the affective symptoms factor.

**Table 3:** Item factor loadings of the four-factorial solution with informed priors and near-zero cross loadings.

	Estimate	SD	*p*-value (one-tailed)	95% CI	
				Lower 2.5%	Upper 2.5%
**Affective symptoms**					
Item 1	**0.807**	0.023	<0.001	0.760	0.849
Item 2	**0.796**	0.027	<0.001	0.738	0.844
Item 3	**0.847**	0.023	<0.001	0.799	0.887
Item 4	**0.826**	0.024	<0.001	0.774	0.869
Item 5	0.064	0.116	0.285	−0.179	0.284
Item 6	−0.107	0.129	0.195	−0.374	0.133
Item 7	−0.069	0.115	0.273	−0.16	0.293
Item 8	0.066	0.165	0.348	−0.265	0.376
Item 9	0.126	0.156	0.216	−0.206	0.402
Item 10	−0.017	0.179	0.462	−0.371	0.330
Item 11	−0.001	0.138	0.497	−0.277	0.264
Item 12	0.007	0.136	0.479	−0.261	0.274
Item 13	0.076	0.119	0.263	−0.169	0.301
**Rumination**					
Item 5	**0.845**	0.094	<0.001	0.676	1.053
Item 6	**0.933**	0.105	<0.001	0.745	1.158
Item 7	**0.866**	0.093	<0.001	0.692	1.058
**Behavioral symptoms**					
Item 8	**0.723**	0.125	<0.001	0.515	0.996
Item 9	**0.602**	0.132	<0.001	0.372	0.891
Item10	**0.823**	0.135	<0.001	0.586	1.112
**Anxiety about personal impact**					
Item 11	**0.876**	0.093	<0.001	0.717	1.082
Item 12	**0.897**	0.089	<0.001	0.740	1.088
Item 13	**0.705**	0.085	<0.001	0.558	0.894

*N* = 486; CI, confidence interval; SD, standard deviation. Values in bold indicate significant estimates (*p* < 0.001).

The intercorrelations of the latent factors were high. Affective symptoms correlated with rumination (0.79), behavioral symptoms (0.74), and anxiety about personal impact (0.65). Further correlations were: rumination with behavioral symptoms (0.55) and anxiety about personal impact (0.61); behavioral symptoms with anxiety about personal impact (0.43).

To examine the effects of informed priors for factor loadings on model estimates, we compared the results of the four-factorial model with informed priors with the model parameters that resulted based on diffuse priors for factor loadings with normal distribution. Differences in main factor loadings and factor covariances were, according to Depaoli and van de Schoot (2017), moderate (1–10%). But the levels of deviation were larger for the cross-loadings of all items on the affective symptoms factor although their model parameters were modeled as diffuse in both analyses. In the alternative model without informed priors, particularly the items 7 (“Unable to stop thinking about losses to the environment”), 9 (“Difficulty enjoying social situations with family and friends”), and 13 (“Feeling anxious that your personal behaviors will do little to help fix the problem”) had stronger than near-zero (>0.1) loadings on the affective symptoms factor, with l_7_ = 0.16, l_9_ = 0.19, and l_13_ = 0.11. Thus, prior assumptions about factor loadings slightly affected the analyses but the conclusions regarding the number and structure of factors remained unchanged.

### 3.3. Association between the HEAS subscales and the DASS-21 in sample 1

Scores of the DASS-21 depression (*n* = 158, *M*[*SD*] = 4.97 [4.41]), anxiety (3.01 [3.67]), and stress subscales (6.32 [4.32]) were below clinical thresholds. A multiple regression model investigating unique associations of each HEAS subscale and the DASS-21 anxiety subscale showed that the affective symptoms subscale (standardized β = 0.29, *p* = 0.011) and the behavioral symptoms subscale (standardized β = 0.25, *p* = 0.009) were significantly associated. Bivariate correlations between all HEAS subscales and all DASS-21 subscales are reported in Table 4.

**Table 4:** Correlations between HEAS subscales and DASS-21 subscales and emotional reactions towards the climate crisis.

	HEAS affective symptoms subscale	HEAS rumination subscale	HEAS behavioral symptoms subscale	HEAS anxiety about personal impact subscale
DASS-21 anxiety subscale	0.41***	0.23**	0.40***	0.28***
DASS-21 depression subscale	0.46***	0.27***	0.49***	0.28***
DASS-21 stress subscale	0.43***	0.23**	0.42***	0.29***
**Emotional reactions to the climate crisis**				
Anxious	0.47***	0.27***	0.16***	0.25***
Distressed	0.40***	0.25***	0.21***	0.32***
Worried	0.38***	0.23***	0.19***	0.31***
Sad	0.38***	0.29***	0.24***	0.34***
Angry	0.37***	0.29***	0.24***	0.31***
Powerless	0.26***	0.12**	0.09*	0.16***
Helpless	0.36***	0.22***	0.16***	0.28***
Ashamed	0.35***	0.25***	0.26***	0.26***
Desperate	0.52***	0.38***	0.28***	0.33***
Hurt	0.40***	0.31***	0.34***	0.24***
Depressed	0.46***	0.28***	0.27***	0.34***
Frustrated	0.40***	0.30***	0.20***	0.38***
Disgusted	0.38***	0.28***	0.24***	0.24***
Guilty	0.32***	0.24***	0.26***	0.35***
Indifferent	−0.09	−0.08	0.04	−0.10*
Calm	−0.23***	−0.15***	−0.01	−0.20***
Optimistic	−0.08	0.03	0.01	−0.01
Confident	−0.09	0.02	0.03	−0.10*

Estimated correlations based on bivariate Pearson correlation coefficients. Data for DASS-21 was only available for sample 1 (*n* = 158). DASS-21, Depression, Anxiety, Stress Scale 21; HEAS, Hogg Eco-Anxiety Scale; *** *p* < 0.001; ** *p* < 0.01; * *p* < 0.05.

### 3.4. Associations between emotional reactions towards the climate crisis and the HEAS affective symptoms subscale

When entering all 18 emotional reactions towards the climate crisis into the multiple linear regression model to test unique relationships with the HEAS affective symptoms subscale, only the emotions “anxious” (standardized β = 0.19, *p* < 0.001), “desperate” (standardized β = 0.19, *p* = 0.001), and “hurt” (standardized β = 0.19, *p* < 0.001) were significantly related when controlling for all other emotions. See Table 4 for bivariate correlations between all HEAS subscales and emotional reactions.

## 4. Discussion

In this study, we translated the original English HEAS into German and assessed its psychometric properties. We measured the internal consistency and investigated whether our data supported the original four-factorial model of the scale. Furthermore, we assessed associations of the HEAS with emotional reactions towards the climate crisis and general depression, anxiety, and stress. In line with the original English HEAS (Hogg et al., 2021), a replication study (Hogg et al., 2023), and recent translations into Turkish (Uzun et al., 2022) and Portuguese (Sampaio, n.d.), the German HEAS showed good reliability (internal consistency) and construct validity, confirming the multidimensional nature of the construct. Results of the Bayesian CFA indicated a good model fit for the four-factorial solution with minor cross-loadings of items from the rumination, behavioral symptoms and anxiety about personal impact factors on the affective symptoms factor. Only three items (item 7: “Unable to stop thinking about losses to the environment”; item 9: “Difficulty enjoying social situations with family and friends”; item 13: “Feeling anxious that your personal behaviors will do little to help fix the problem”) showed minor (>0.1) cross-loadings on the affective symptoms factor. Moreover, the four-factorial model showed a better fit to the data than the three-, two-, and one-factorial solutions. An additional conventional CFA supported the good model fit of the four-factorial model. In summary, we were able to reproduce the four-factorial structure of the original English version for the German translation of the HEAS.

Similar to the results of Hogg et al. (2021), correlations between subscales of the German HEAS and the DASS-21 ranged from weak to moderate, indicating that the dimensions of eco-anxiety were distinct from general depression, anxiety, and stress, but shared a significant proportion of variance. This is in line with prior work reporting weak to moderate but consistent positive associations between negative eco-emotions and poor mental health (Stanley et al., 2021; Stewart, 2021; Ogunbode et al., 2023). In a multiple linear regression model only the affective and behavioral symptoms subscales of the HEAS were related to general anxiety, emphasizing the multidimensional properties of eco-anxiety. Moderate correlations between eco-anxiety and other negative, but not positive emotions suggest that engaging with the climate and environmental crises simultaneously evokes a variety of negative emotions, as proposed by other studies (Hickman et al., 2021; Ojala et al., 2021). A multiple linear regression model of 18 emotions on the affective symptoms subscale showed that only the emotions anxious, desperate and hurt uniquely predicted the affective symptoms subscale which supports the concurrent and discriminant validity of this subscale.

The current study contributes to a growing international research interest in understanding the emotional consequences of the climate and environmental crises and highlights that eco-anxiety is more complex than just feeling anxious or concerned. Rather, eco-anxiety comprises a complex and intertwined set of ruminations, potential impairments, and deep concerns. Saying that, however, the simple solution of “reducing anxiety” that is usually a goal in the treatment of anxiety disorders may not prove fruitful in the case of eco-anxiety. In contrast to anxiety disorders such as agoraphobia, eco-anxiety is not an inadequate or exaggerated reaction to an objectively harmless situation because the dimensions of the climate and environmental crises are overwhelming, and the threat is real. Thus, in most cases, eco-anxiety can be seen as a reasonable response to these excessive crises (Heinzel, 2022). Further research will be required to define cut-off values identifying severe cases of eco-anxiety (e.g., when professional support to deal with the anxiety would be recommended). Quantifying the level of impairment and frequency of symptoms as done in the HEAS is a good starting point for this line of research. We anticipate that climate-and eco-anxiety will increase globally as the climate and environmental crises unfold in the upcoming years. Thus, precisely operationalizing these constructs and developing reliable and valid scales is important not only for research but also for practice. Having adequate measures can increase awareness for these types of anxiety in society and potentially support clinicians to identify severe cases that may need help.

Investigating behavioral consequences of eco-anxiety including the relationship between eco-anxiety and pro-environmental behavior is another important line of research. Prior work suggests that the experience of climate- or eco-anxiety is often associated with motivation for pro-environmental action and policy support (Wullenkord et al., 2021; Whitmarsh et al., 2022; Ogunbode et al., 2023). However, further research is required to understand under which conditions this anxiety transforms into action, rather than into cognitive suppression/ assimilation or emotional apathy (Lamb et al., 2020). We believe that eco-anxiety can be overcome if comprehensive and effective measures to counteract the climate crisis and protect the environment will be implemented worldwide (Heinzel, 2022).

### 4.1. Limitations and future perspectives

Some limitations need to be considered when interpreting our results. First, the study samples were not representative for the general population given that large proportions of the sample were young, female and highly educated which is due to recruitment in university settings. Thus, future research should be based on representative samples to assess whether our results can be generalized to the German population. Second, while we were able to investigate several important psychometric properties of the German HEAS in this study, other properties such as test–retest reliability remain to be investigated in future studies (Mokkink et al., 2010).

Finally, given the confirmatory approach of the current study, other possible conceptions of eco-anxiety were not investigated. As suggested by Hogg et al. (2021), eco-anxiety may appear differently in groups or societies already more strongly affected by the negative consequences of the climate and environmental crises, highlighting the need for further research on eco-anxiety in the Global South, indigenous people and other exposed populations such as farmers. Moreover, future work should investigate the overlap between eco-anxiety and related emotions including, but not limited to, the anxiety of societal impacts on the environment - as opposed to personal impacts - as well as frustration and anger about a lack of effective policies.

### 4.2. Conclusion

The German translation of the HEAS was tested in a sample of 486 participants in Germany and the scale and its subscales were found to be as reliable as the original English version. The Bayesian CFA confirmed the multidimensionality of the construct supporting a four-factorial model of eco-anxiety (affective symptoms, rumination, behavioral symptoms, anxiety about personal impact). Regression results and weak to moderate associations between the HEAS subscales and the DASS-21 subscales indicate that eco-anxiety is distinct from general depression, anxiety, and stress, but shares a significant proportion of variance. Taken together, the study suggests that the German HEAS is a reliable and valid scale to measure eco-anxiety.

## Data availability statement

The raw data supporting the conclusions of this article will be made available by the authors, without undue reservation.

## Ethics statement

The studies involving humans were approved by local ethics committee of Freie Universität Berlin, Berlin, Germany. The studies were conducted in accordance with the local legislation and institutional requirements. The participants provided their written informed consent to participate in this study.

## Author contributions

SH, FS, GR, FP, JK, and ME contributed to conception and design of the study. SH, MS-H, and FP translated the HEAS scale. MT, MS-H, FS, CP, and JK coordinated the study administration and data acquisition. SH, MS-H, and MB performed the statistical analyses. SH and MB wrote the first draft of the manuscript. GR, FP, and ME wrote sections of the manuscript. All authors contributed to the article and approved the submitted version.

## Conflict of interest

The authors declare that the research was conducted in the absence of any commercial or financial relationships that could be construed as a potential conflict of interest.

## Publisher’s note

All claims expressed in this article are solely those of the authors and do not necessarily represent those of their affiliated organizations, or those of the publisher, the editors and the reviewers. Any product that may be evaluated in this article, or claim that may be made by its manufacturer, is not guaranteed or endorsed by the publisher.

## References

[ref1] Asparouhov T. Muthén B. (2017). Prior-posterior predictive p-values (Mplus Web Notes: No. 22). Available at: https://www.statmodel.com/download/PPPPvalues.pdf (Accessed September 7, 2023).

[ref2] Bentler P. M. (1990). Comparative fit indexes in structural models. Psychol. Bull. 107, 238–246. doi: 10.1037/0033-2909.107.2.238 2320703

[ref3] Brislin R. W. (1970). Back-translation for cross-cultural research. J. Cross-Cult. Psychol. 1, 185–216. doi: 10.1177/135910457000100301

[ref4] Cain M. K. Zhang Z. (2019). Fit for a Bayesian: an evaluation of PPP and DIC for structural equation modeling. Struct. Equ. Model. Multidiscip. J. 26, 39–50. doi: 10.1080/10705511.2018.1490648

[ref5] Clayton S. (2020). Climate anxiety: psychological responses to climate change. J. Anxiety Disord. 74:102263. doi: 10.1016/j.janxdis.2020.102263 32623280

[ref6] Clayton S. Karazsia B. T. (2020). Development and validation of a measure of climate change anxiety. J. Environ. Psychol. 69:101434. doi: 10.1016/j.jenvp.2020.101434

[ref7] Corvalan C. Gray B. Prats E. V. Sena A. Hanna F. Campbell-Lendrum D. (2022). Mental health and the global climate crisis. Epidemiol. Psychiatr. Sci. 31:e86. doi: 10.1017/S2045796022000361, PMID: 36459133 PMC9762138

[ref8] Depaoli S. (2021). Bayesian Structural Equation Modeling. New York, NY: The Guilford Publications.

[ref9] Depaoli S. van de Schoot R. (2017). Improving transparency and replication in Bayesian statistics: the WAMBS-checklist. Psychol. Methods 22, 240–261. doi: 10.1037/met0000065 26690773

[ref10] Dorer B. (2018). ESS round 9 translation guidlines. Mannheim: European Social Survey GESIS.

[ref11] Gelman A. Rubin D. B. (1992a). A single series from the Gibbs sampler provides a false sense of security. Bayesian Stat. 4, 625–631.

[ref12] Gelman A. Rubin D. B. (1992b). Inference from iterative simulation using multiple sequences. Stat. Sci. 7, 457–472. doi: 10.1214/ss/1177011136

[ref13] Heinzel S. (2022). “Klima-Angst – Eine Angemessene Reaktion auf Eine Maßlose Krise?,” in Climate Emotions - Klimakrise und Psychische Gesundheit. Eds. K. van Bronswijk and C. Hausmann (Gießen: Psychosozial-Verlag).

[ref14] Helm S. V. Pollitt A. Barnett M. A. Curran M. A. Craig Z. R. (2018). Differentiating environmental concern in the context of psychological adaption to climate change. Glob. Environ. Chang. 48, 158–167. doi: 10.1016/j.gloenvcha.2017.11.012

[ref15] Hickman C. Marks E. Pihkala P. Clayton S. Lewandowski R. E. Mayall E. E.. (2021). Climate anxiety in children and young people and their beliefs about government responses to climate change: a global survey. Lancet Planet. Health 5, e863–e873. doi: 10.1016/S2542-5196(21)00278-3, PMID: 34895496

[ref16] Hogg T. L. Stanley S. K. O’Brien L. V. (2023). Synthesising psychometric evidence for the climate anxiety scale and Hogg eco-anxiety scale. J. Environ. Psychol. 88:102003. doi: 10.1016/j.jenvp.2023.102003

[ref17] Hogg T. L. Stanley S. K. O’Brien L. V. Wilson M. S. Watsford C. R. (2021). The Hogg eco-anxiety scale: development and validation of a multidimensional scale. Glob. Environ. Chang. 71:102391. doi: 10.1016/j.gloenvcha.2021.102391

[ref18] Hu L. Bentler P. M. (1999). Cutoff criteria for fit indexes in covariance structure analysis: conventional criteria versus new alternatives. Struct. Equ. Model. Multidiscip. J. 6, 1–55. doi: 10.1080/10705519909540118

[ref19] Kates R. W. Travis W. R. Wilbanks T. J. (2012). Transformational adaptation when incremental adaptations to climate change are insufficient. Proc. Natl. Acad. Sci. 109, 7156–7161. doi: 10.1073/pnas.1115521109, PMID: 22509036 PMC3358899

[ref20] Lamb W. F. Mattioli G. Levi S. Roberts J. T. Capstick S. Creutzig F.. (2020). Discourses of climate delay. Glob. Sustain. 3:e17. doi: 10.1017/sus.2020.13

[ref21] Larionow P. Sołtys M. Izdebski P. Mudło-Głagolska K. Golonka J. Demski M.. (2022). Climate change anxiety assessment: the psychometric properties of the polish version of the climate anxiety scale. Front. Psychol. 13:870392. doi: 10.3389/fpsyg.2022.870392, PMID: 35645848 PMC9130850

[ref22] Lovibond P. Lovibond S. (1995). DASS 21= depression, anxiety stress scales. The structure of negative emotional states: comparison of the depression anxiety stress scales (DASS) with the Beck depression and anxiety inventories. Behav. Res. Ther. 33, 335–343. doi: 10.1016/0005-7967(94)00075-U, PMID: 7726811

[ref23] Mokkink L. B. Terwee C. B. Patrick D. L. Alonso J. Stratford P. W. Knol D. L.. (2010). The COSMIN study reached international consensus on taxonomy, terminology, and definitions of measurement properties for health-related patient-reported outcomes. J. Clin. Epidemiol. 63, 737–745. doi: 10.1016/j.jclinepi.2010.02.006, PMID: 20494804

[ref24] Mouguiama-Daouda C. Blanchard M. A. Coussement C. Heeren A. (2022). On the measurement of climate change anxiety: French validation of the climate anxiety scale. Psychol Belg 62, 123–135. doi: 10.5334/pb.1137, PMID: 35414943 PMC8954884

[ref25] Muthén B. Asparouhov T. (2012). Bayesian structural equation modeling: a more flexible representation of substantive theory. Psychol. Methods 17, 313–335. doi: 10.1037/a0026802, PMID: 22962886

[ref26] Muthén L. K. Muthén B. (2017). Mplus User’s Guide: Statistical Analysis with Latent Variables, User’s Guide. 8th edn. Los Angeles, CA: Muthén & Muthén.

[ref27] Ogunbode C. A. Pallesen S. Böhm G. Doran R. Bhullar N. Aquino S.. (2023). Negative emotions about climate change are related to insomnia symptoms and mental health: cross-sectional evidence from 25 countries. Curr. Psychol. 42, 845–854. doi: 10.1007/s12144-021-01385-4

[ref28] Ojala M. Cunsolo A. Ogunbode C. A. Middleton J. (2021). Anxiety, worry, and grief in a time of environmental and climate crisis: a narrative review. Annu. Rev. Environ. Resour. 46, 35–58. doi: 10.1146/annurev-environ-012220-022716

[ref29] Pihkala P. (2020). Eco-anxiety and environmental education. Sustainability 12:10149. doi: 10.3390/su122310149

[ref30] Sampaio (n.d.). Validating a Measure of Eco-Anxiety with Portuguese Young Adults and Exploring Associations with Environmental Action. [under review]. 10.1186/s12889-023-16816-z PMC10546781 37784133

[ref31] Searle K. Gow K. (2010). Do concerns about climate change lead to distress? Int. J. Climate Change Strateg. Manage. 2, 362–379. doi: 10.1108/17568691011089891

[ref32] Simon P. D. Pakingan K. A. Aruta J. J. B. R. (2022). Measurement of climate change anxiety and its mediating effect between experience of climate change and mitigation actions of Filipino youth. Educ. Dev. Psychol. 39, 17–27. doi: 10.1080/20590776.2022.2037390

[ref33] Stanley S. K. Hogg T. L. Leviston Z. Walker I. (2021). From anger to action: differential impacts of eco-anxiety, eco-depression, and eco-anger on climate action and wellbeing. J. Climate Change Health 1:100003. doi: 10.1016/j.joclim.2021.100003

[ref35] Steiger J. H. (1980). Statistically Based Tests for the Number of Common Factors. Paper presented at the Annual Meeting of the Psychometric Society, Iowa City.

[ref36] Stewart A. E. (2021). Psychometric properties of the climate change worry scale. Int. J. Environ. Res. Public Health 18:494. doi: 10.3390/ijerph18020494, PMID: 33435348 PMC7826965

[ref37] Tam K.-P. Chan H.-W. Clayton S. (2023). Climate change anxiety in China, India, Japan, and the United States. J. Environ. Psychol. 87:101991. doi: 10.1016/j.jenvp.2023.101991

[ref38] Tucker L. R. Lewis C. (1973). A reliability coefficient for maximum likelihood factor analysis. Psychometrika 38, 1–10. doi: 10.1007/BF02291170

[ref39] Uzun K. Öztürk A. F. Karaman M. Cebeci F. Altin M. O. Arici A.. (2022). Adaptation of the eco-anxiety scale to Turkish: a validity and reliability study. CAL 9, 110–115. doi: 10.54614/ArcHealthSciRes.2022.21151

[ref40] Whitmarsh L. Player L. Jiongco A. James M. Williams M. Marks E.. (2022). Climate anxiety: what predicts it and how is it related to climate action? J. Environ. Psychol. 83:101866. doi: 10.1016/j.jenvp.2022.101866

[ref41] Wullenkord M. Tröger J. Hamann K. R. S. Loy L. Reese G. (2021). Anxiety and Climate Change: A Validation of the Climate Anxiety Scale in a German-Speaking Quota Sample and an Investigation of Psychological Correlates. Climatic Change 168, 1–23. doi: 10.1007/s10584-021-03234-6

